# Intraoperative Fluid Restriction in Pancreatic Surgery: A Double Blinded Randomised Controlled Trial

**DOI:** 10.1371/journal.pone.0140294

**Published:** 2015-10-14

**Authors:** Ganapathy van Samkar, Wietse J. Eshuis, Roelof J. Bennink, Thomas M. van Gulik, Marcel G. W. Dijkgraaf, Benedikt Preckel, Stefan de Hert, Dirk J. Gouma, Markus W. Hollmann, Olivier R. C. Busch

**Affiliations:** 1 Department of Anesthesiology, Academic Medical Center, Amsterdam, the Netherlands; 2 Department of Surgery, Academic Medical Center, Amsterdam, the Netherlands; 3 Department of Nuclear Medicine, Academic Medical Center, Amsterdam, the Netherlands; 4 Clinical Research Unit, Academic Medical Center, Amsterdam, the Netherlands; 5 Department of Anesthesiology, University of Ghent, Ghent, Belgium; San Raffaele Scientific Institute, ITALY

## Abstract

**Background:**

Perioperative fluid restriction in a variety of operations has shown improvement of: complications, recovery of gastrointestinal function and length of stay (LOS). We investigated effects of crystalloid fluid restriction in pancreatic surgery. Our hypothesis: enhanced recovery of gastrointestinal function.

**Methods:**

In this double-blinded randomized trial, patients scheduled to undergo pancreatoduodenectomy (PD) were randomized: standard (S:10ml/kg/hr) or restricted (R:5ml/kg/hr) fluid protocols. Primary endpoint: gastric emptying scintigraphically assessed on postoperative day 7.

**Results:**

In 66 randomized patients, complications and 6-year survival were analyzed. 54 patients were analyzed in intention to treat: 24 S-group and 30 R-group. 32 patients actually underwent a PD and 16 patients had a palliative gastrojejunostomy bypass operation in the full protocol analysis. The median gastric emptying time (T½) was 104 minutes (S-group, 95% confidence interval: 74–369) versus 159 minutes (R-group, 95% confidence interval: 61–204) (*P* = 0.893, NS). Delayed gastric emptying occurred in 10 patients in the S-group and in 13 patients in the R-group (45% and 50%, P = 0.779, NS). The primary outcome parameter, gastric emptying time, did not show a statistically significant difference between groups.

**Conclusion:**

A fluid regimen of 10ml/kg/hr or 5ml/kg/hr during pancreatic surgery did not lead to statistically significant differences in gastric emptying. A larger study would be needed to draw definite conclusions about fluid restriction in pancreatic surgery.

**Trial registration:**

ISRCTN62621488

## Introduction

Perioperative fluid restriction can enhance recovery of gastrointestinal function, reduce complications and also hospital stay in patients subjected to a variety of abdominal surgical procedures [1–6]. This beneficial effect of fluid restriction is due to a decrease in visceral and interstitial edema caused by crystalloids infusion during surgery [7–9]. The timing of the fluids is essential to the outcome of surgery [10]. A study in colorectal surgery demonstrated a lack of effect on length of stay, while reducing complications [11]. Other studies however, focusing on other types of surgery, failed to report beneficial effects of fluid restriction [4, 12, 13]. A recent meta-analysis concluded that perioperative fluid restriction does not reduce complications or length of hospital stay [14]. Most studies concerning fluid restriction use inaccurate definitions, “restricted” ranging from 4 to 9 ml/kg/hr and “liberal” from 9 to 18 ml/kg/hr. Great variation exists in composition of fluids and other measures used: glucose 5%, saline 0.9%, saline 0.18% + dextrose 4%, Ringers Lactate, starch, furosemide for weight control etcetera [8, 15, 16]. This effect of fluid restriction has not been studied in pancreatic surgery, although a study on acute normovolemic hemodilution (ANH) in pancreatoduodenectomy (PD) found more anastomotic complications in the ANH-group—a finding which, according to the authors, could be explained by the associated greater fluid administration in this study arm [17]. A recent (retrospective) study of patients after pancreatic resection found no significant correlation between complications and the intraoperative amount of fluids [18]. A procedure-specific approach, with procedure-specific endpoints, is necessary to determine the best method of perioperative fluid management for each surgical procedure [8, 19].

Many trials have compared the difference in clinical outcome between restricted and liberal fluid regimens perioperatively. To separate effects of *post*operative restriction and *intra*operative restriction different kinds of trials are needed. An earlier trial in our hospital concerning fluid restriction in the postoperative phase after major abdominal surgery was discontinued because of a significantly higher number of complications in the fluid restricted group [20]. Surgical handling combined with fluid overload could have its maximal effect in the intraoperative phase, which is the reason we conducted this randomized controlled trial focusing on fluid restriction intraoperatively. The aim of this study was to show the effect of a restricted, intraoperative crystalloid fluid protocol on gastric emptying, postoperative complications and duration of hospital stay, as compared to our standard intraoperative fluid protocol, in patients with a suspected pancreatic tumor, undergoing an exploration for resection. Our hypothesis was that intraoperative crystalloid fluid restriction would have a beneficial effect on gastric emptying, analogous to an earlier study by Lobo and co-authors [3].

## Materials and Methods

### Study design and patients

The study was designed as a prospective, double blinded, single center randomized controlled trial (EPOR trial, ISRCTN62621488), for which approval of the AMC Medical Ethics Committee was obtained. (S1 Protocol) Written informed consent was obtained from all participating patients. The setting was at the Academic Medical Center/University of Amsterdam, in Amsterdam, The Netherlands. All operations were performed by a selected team of 3 experienced hepato-pancreato-biliary surgeons, who performed at least 100 pancreatic resections on annual basis.

Inclusion criteria for study participation were: scheduled to undergo pancreatoduodenectomy (PD) for a suspected pancreatic head or periampullary tumor, age >18 years and American Society of Anesthesiologists (ASA) classification I-IV. Exclusion criteria were: diabetes mellitus, renal failure, drug or alcohol abuse, clinical signs of preoperative gastroparesis, unfit to participate due to language or psychiatric problems, contraindications for epidural (such as severe coagulation disorder), blood loss of more than 50% of circulating volume and/or postoperative ICU, or participation in another clinical trial. Patients were also excluded from further analysis in the case of preoperative or early intraoperative protocol violation: for example when during the operation no (pylorus preserving) PD or gastric bypass was performed, or in case of failure of epidural catheter placement, to minimize the effect of postoperative intravenous opiates on gastric emptying.

Time period of inclusion was: 1^st^ May 2006 to 1^st^ March 2009. Follow-up time was planned for at least a year. Inclusion of the patients was done preferably at least 7 days before the planned operation date by a researcher not involved with the study, giving patients reasonable time to think about participation. An epidemiologist provided a computer generated randomization chart on paper, which was kept by a researcher not involved with the study, and checked by an independent noninvolved researcher. Participating patients were randomly assigned (1:1 ratio) to either a restricted or a standard intraoperative fluid management. Neither the anesthesiologist nor the operating surgeon was aware of the randomization group, until the completion of the entire study. This was achieved by a covered volumetric infusion pump, with crystalloid infusion fluid rate set by a research assistant. Under an opaque black plastic clothes bag, five bags of 500 ml were connected to each other, containing Ringers Lactate, totaling 2500 ml, and in turn connected to an infusion pump as above. This was repeated if necessary by an independent researcher, if the infusion pump was almost empty during the procedure. That way, the amount of intravenous fluids was not known to the anesthesiologist, who only administered extra colloids, red blood cells and medication as indicated in protocol (blood loss was compensated in equal volume by colloid infusion in both groups, red blood cells were given when hemoglobin values decreased below 5.0 mmol/l).

#### Study procedures

Antithrombotic and antibiotic prophylactic medication (intravenous cefuroxime and clindamycin) was administered in all patients. No prophylactic prokinetic drugs were given. The intended standard surgical procedure was a pylorus-preserving PD with removal of lymph nodes at the right side of the portal vein, as described earlier [21]. 32 patients actually underwent a PD. 16 patients underwent a palliative bypass procedure; these were included in the analysis. Patients who did not undergo either PD or palliative bypass were excluded from analysis. In our study these patients were: 2 patients with bile duct resection, 1 patient with a benign pancreatic cyst, 1 patient with pancreatic corpus tumor, and in 1 patient we were unable to perform even a palliative bypass (“open-close surgery”).

Anesthesiological management included a thoracic (T7-T8 or T8-T9) epidural catheter. Epidural infusion consisted of bupivacaine 0.25% with sufentanil 0.5 microgram per ml at a speed of 8–10 ml per hour in both groups. In case of a failed placement of the epidural catheter, patients received intravenous patient controlled analgesia (PCA) with morphine postoperatively and were excluded from further study participation due to the influence of morphine on gastric emptying. FiO2 was 60%.[22, 23] If mean arterial pressure (MAP) was ≥ 20% below baseline, norepinephrine (standard dose: 1–4 μg/kilogram body weight/hr) was used (in the absence of blood loss) to keep MAP at +/- 20% of its baseline value. Isoflurane was used in both groups at endtidal concentrations of 1 MAC (age corrected minimum alveolar concentration.)

#### Fluid management

Standard and restricted fluid infusions (S group and R group) consisted of 10 ml/kg/hr (S) and 5 ml/kg/hr (R) Ringer’s Lactate. This was an arbitrary decision, based on earlier studies intraoperative fluid administration in abdominal surgery [24, 25]. With a predicted average operating time of 6 hours, this would be 4800 ml or 2400 ml respectively in a patient of 80 kg.

All patients were monitored overnight in the post anesthesia care unit (PACU) and were discharged to the surgical ward the following day. Post-operative fluid management was standardized and similar in both groups. Differences between groups in fluid administration were only in the intraoperative period. The total daily fluid intake was set at 2500 ml/24 hrs, including any oral intake, with correction for nasogastric and other drain loss. The nasogastric drain was removed if production was below 300 ml/24 hrs.

#### Outcome measures

The primary outcome measure was gastric emptying time (i.e. reduction of minutes needed to achieve a 50% emptying of the stomach), as measured by scintigraphy with a solid test meal on the seventh postoperative day. Secondary outcome parameters included postoperative surgical complications, including the incidence of delayed gastric emptying (DGE) according to the definition of the International Study Group of Pancreatic Surgery (ISGPS), relaparotomy rate, non-surgical complications, mortality, duration of hospital stay, values of urea, creatinine, albumin, remaining length of duodenum and body weight [26]. We had originally planned to include nutritional intake (kCal/day) as well, but had to exclude this parameter because of the lack of possibilities of the nursing department to document this particular parameter in a detailed way.

Solid phase scintigraphy was performed on two days: 1. On the day preceding the operation, to establish a baseline measurement. 2. On the seventh postoperative day, using a small size 75-gram pancake labeled with 10 MegaBecquerel technetium 99m colloid, with a caloric content of 576 kJ (137 kCal), containing 70% carbohydrates, 14% protein, and 16% fat. Patients ingested at least 75% of the meal with a glass of water within 5 minutes (min). Patients did not receive any prokinetic drugs 24 hours prior to the gastric emptying examination [27].

#### Sample size

A difference in gastric emptying time of 30 min between the study groups was considered as statistically significant, as normal solid phase emptying has been shown to fall in the range of approximately 60 min in volunteers [3, 28]. We calculated that 22 patients in each group would have 90% power to detect a difference in mean emptying time of 30 min, assuming that the common standard deviation was 33 min and using a two-group Student’s *t*-test with a 0.05 one-sided significance level.

#### Statistical analysis

All clinically relevant outcome measures were analyzed by the intention-to-treat principle. Gastric emptying was only analyzed in patients who underwent gastric emptying scintigraphy (per-protocol population). Data are described as means with standard deviation or medians with interquartile ranges where appropriate. Continuous data were analyzed by the Student’s *t*-test or Mann-Whitney U test, depending on the distribution of the data. The Chi-square test or Fisher’s exact test (in case of zero cell counts) was used to compare categorical data. A *P*-value below 0.05 was considered statistically significant. All analyses were performed with SPSS Version 22.0 (SPSS Inc., Chicago, IL, USA).

## Results

### Trial profile

**Fig 1:**
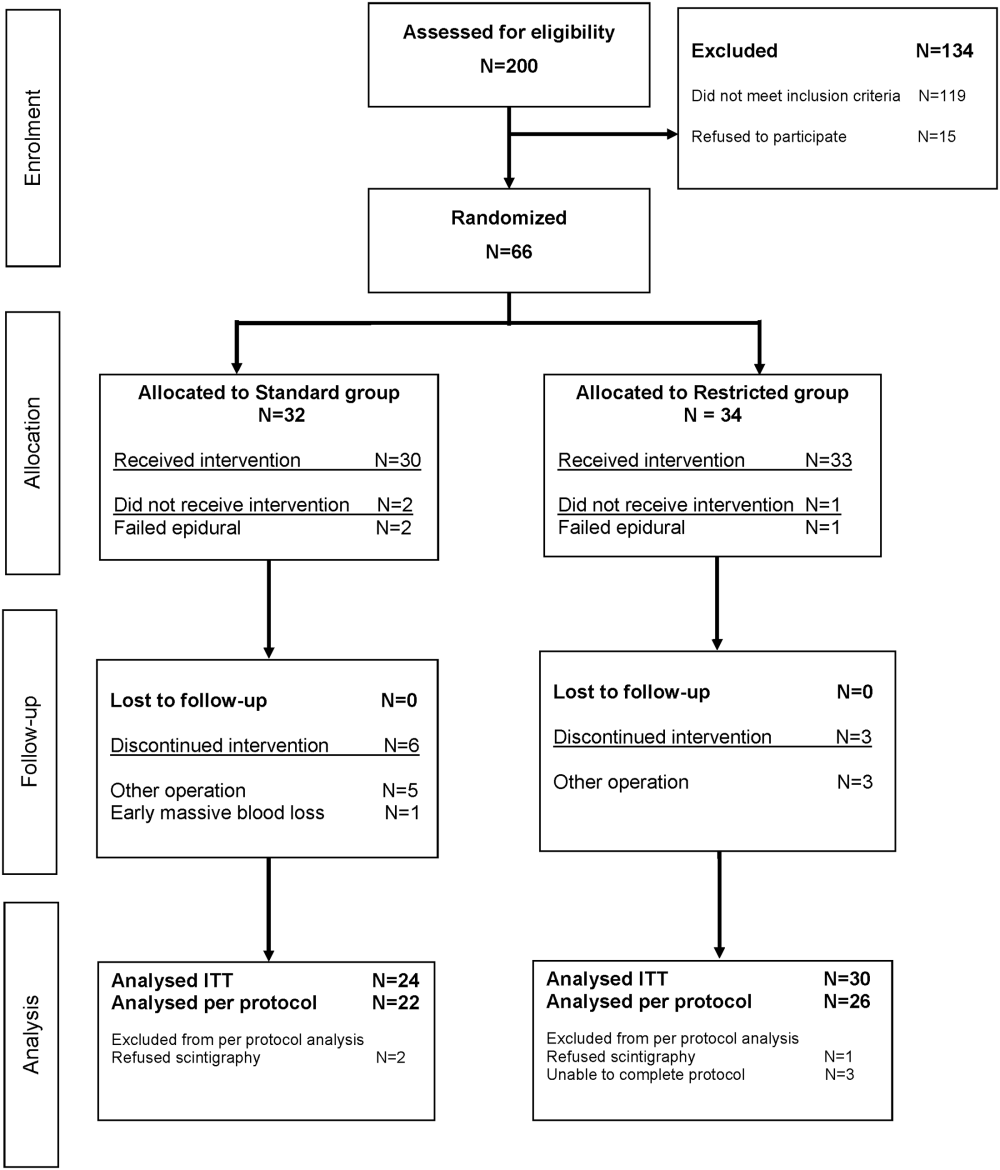
Trial flow chart.

**Fig 2:**
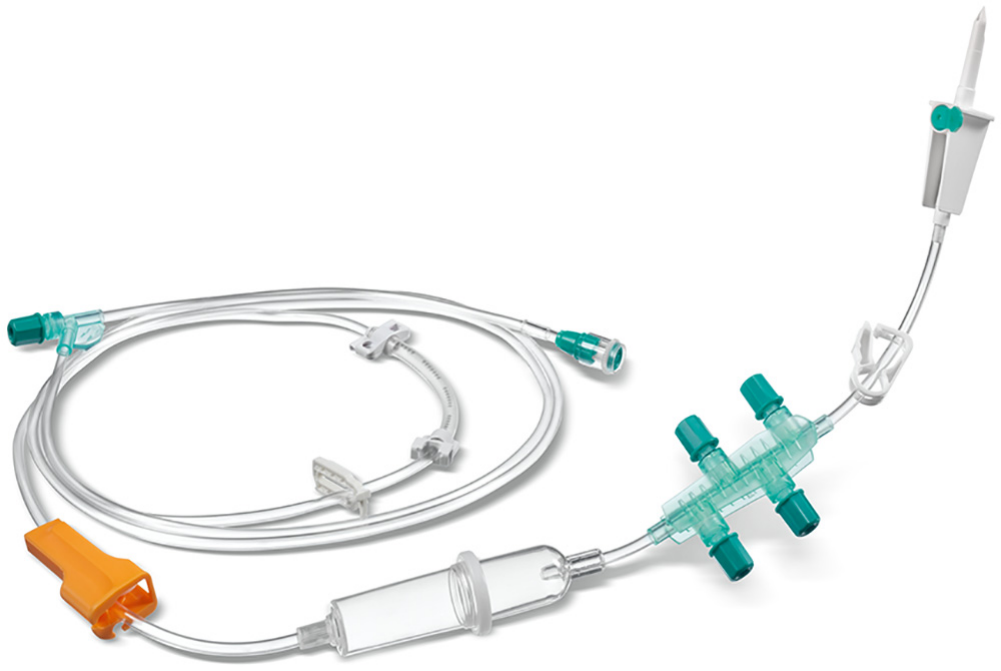
Connection piece. Cyto Set system enabling simultaneous use of 5 infusion bags.

The trial profile is shown in the trial flow chart (Fig 1). We randomized 66 patients. In the standard group 32 patients were allocated. Of these, 2 had a failed epidural. In 5 patients a different operation was performed than originally planned (not a pancreatoduodenectomy or bypass). In 1 patient, there was massive blood loss at an early stage during the operation, resulting in loss of more than 50% of circulating volume, and making it impossible to follow protocolled fluid infusion. We excluded these patients. We allocated 34 patients to the restricted group. In the restricted group, 1 patient had a failed epidural. In 3 patients, the final operation differed from the planned one (not a pancreatoduodenectomy or bypass). The above resulted in 54 patients (24 patients in the S group, 30 in the R group) to be analyzed in the intention-to-treat analysis. Complications and 6-year survival are analyzed for 66 patients. In the standard group, 2 patients refused scintigraphy in the postoperative phase. In the restricted group, 1 patient refused scintigraphy, and 3 patients were unable to complete the protocol (second scan) due to logistic reasons (scanning machine was unavailable due to repairs). Postoperative scans were therefore only done for 48 patients. This resulted in 48 patients (22 patients in the S group, 26 in the R group) in the per-protocol analysis. After the initial completion of the calculated 44 patients, we obtained permission from the ethics committee to recruit additional patients, to reach the number of 54 included patients, because dropouts had resulted in an unequal distribution between groups, resulting in a number lower than required by the power calculation, in one of the groups. (S1 File) The infusion system enabling simultaneous connection of 5 infusion bags is shown below. (Fig 2.)

### Patient and treatment characteristics

There were no differences between both groups in age, gender, weight, ASA classification or underlying disease.

Treatment characteristics are shown in Table 1. The mean amount of intraoperative crystalloids infusion was 9.8 ml/kg/hour in the S group, and 5.1 ml/kg/hour in the R group, indicating that the randomization and protocol had been followed properly. The mean amount of colloid administration was 1 liter in the S group, and 1.4 liters in the R group (*P* = 0.029). Norepinephrine dose, blood loss and intraoperative diuresis were similar in both groups. The cumulative 5 day fluid balance includes oral fluid intake, and shows similar balances in both groups. Weight increase was slightly higher in the standard group, but this difference was not significant. Both groups showed a substantial decrease in serum albumin on postoperative day 5, without any significant differences between groups. Creatinine and urea levels showed comparable decreases in both groups.

**Table 1:** Treatment characteristics of 54 patients who underwent PPPD or palliative bypass procedure, and were randomized to the Standard or Restricted group. Data shown include fluid infusion data during the procedure, and i.v.* noradrenalin and diuresis at the end of operation.

Endpoint	Standard (max N = 24)	Restricted (max N = 30)	P-value
**Operation—No. (%)**			
** Resections:**			0.08
** Pylorus preserving PD**	13 (54)	23 (77)	
** Palliative procedures:**			
** Bypass procedure with GJ**	11 (46)	7 (23)	
**Duration of procedure in minutes**	248 (104) [210–288]	289 (78) [262–316]	0.101
**Blood loss in liters** **	1.0 (0.8) [0.6–1.3]	1.1 (0.6) [0.9–1.4]	0.393
**Actual crystalloids during operation in ml/kg/hour** †	9.8 (1.1) [9.3–10.2]	5.1 (0.3) [5.0–5.2]	<0.001
**Actual crystalloids during operation in liters** † §	2.5 (1.7–3.6) [2.0–3.4]	2.0 (1.4–2.4) [1.6–2.2]	0.013
**Actual crystalloids during PPPD in liters** # §	3.4 (2.5–6.0) [2.7–4.9]	2.1 (1.6–2.5) [1.8–2.4]	<0.001
**Colloids during operation in liters** †	1.0 (0.6) [0.8–1.3]	1.4 (0.6) [1.2–1.6]	0.029
**Plasma during operation in ml**	108 (367) [45–271]	195 (423) [70–337]	0.432
**Noradrenalin i.v.** * **in micrograms /kg /min** ˄	0.04 (0.03) [0.03–0.05]	0.05 (0.05) [0.03–0.07]	0.388
**Intraoperative diuresis in ml /kg /hr** ˄˄	1.9 (1.2) [1.5–2.4]	1.5 (1.1) [1.2–2.0]	0.195
**Cumulative 5 day fluid balance** ˜	5.5 (4.2) [3.5–7.7]	4.6 (3.4) [3.0–6.2]	0.495
**Increase in weight on day 5** ǂ **(in Kg)**	5.0 (3.4) [3.3–6.5]	3.5 (3.0) [2.3–4.6]	0.180
**Preoperative serum albumin (mmol/l)** $	44 (3.9) [42–45]	43 (3.5) [41–44]	0.399
**Albumin, on 5** ^**th**^ **postoperative day (mmol/l)** $$	30 (3.0) [29–31]	30 (3.9) [28–31]	0.644
**Preoperative creatinine (mmol/l)** &	67 [62–73]	71 [65–77]	0.329
**Creatinine, on 5** ^**th**^ **postoperative day (mmol/l)** &*	58 [57–59]	61 [56–65]	0.311
**Preoperative serum urea (mmol/l)** &&	5.3 [4.3–6.4]	5.6 [5.1–6.3]	0.330
**Serum urea on 5** ^**th**^ **postoperative day (mmol/l)** &&&	3.5 [3.2–3.7]	3.7 [2.9–4.7]	0.339
**Remaining length of duodenum in cm** ##	1.4 (1.4) [0.8–2.0]	2.3 (1.6) [1.7–2.8]	0.05

Values are shown as mean, (SD), (1^st^ Quartile-3^rd^ Quartile), [Bias-corrected 95% confidence interval] unless otherwise specified.

* i.v., intravenous;

**N: 22 versus 28;

^†^ N: 24 versus 29;

^§^ median and interquartile range;

^#^N:13 versus 23;

^˄^N: 22versus 27;

^˄˄^N: 24 versus 28;

^˜^N: 14 versus 16;

^ǂ^, N = 14 versus 19;

^$^, N = 19 versus 25;

^$$^, N = 21 versus 23;

^&^ median, N = 22 versus 26;

^&^*, N:21 versus 24;

^&&^, N = 20 versus 23;

^&&&^, N = 21 versus 24;

^##^, N = 21 versus 25.

### Gastric emptying scintigraphy (primary endpoint)

The preoperative gastric emptying scans were performed on the day preceding the operation. The distribution of all gastric emptying times showed a skewness of 1.027, making it highly skewed. We have split these preoperative scans into two groups in the table, even though it was not known at the time of scanning which group the patients would be allocated to. Postoperative scans: 48 Patients underwent a gastric scintigraphy on the seventh postoperative day (Table 2). The postoperative gastric emptying times were also not normally distributed: the Shapiro Wilk test showed a difference between a normal distribution and our data with a significance of <0.001, so therefore we calculated difference in medians instead of the planned mean differences in emptying times between groups. The assumed common standard deviation (SD) of 33 minutes turned out to be higher: SD of 143 (standard group) and SD of 137 (restrictive group). The median gastric emptying time (T½) was 104 minutes (S-group, 95% confidence interval: 74–369) versus 159 minutes (R-group, 95% confidence interval: 61–204) (*P* = 0.893, NS). The differences in our primary endpoint are not significant. (Table 2). The number of patients who were judged to have a delay in gastric emptying, based on consensus recommended gastric emptying scintigraphy normal range criteria, was 10 (45%) in the S group and 13 (50%) in the R group (*P* = 0.779, NS) [29]. The median percentage of radioactivity remaining in the stomach after 120 minutes (% RA 120) was 47 in the S group (95% confidence interval: 30–94) and 64 in the R group (95% confidence interval: 35–91) (*P* = 0.852, NS). The primary outcome parameter (gastric emptying time) did not show a statistically significant difference between groups.

**Table 2:** Results of scintigraphy performed preoperatively and 7 days postoperatively in 48 full protocol patients who underwent PPPD or palliative bypass procedure and were randomized to a Standard or a Restricted fluid protocol. The second part specifies 32 patients who only underwent a PPPD procedure and were randomized to a Standardized or a Restricted fluid protocol.

	Per-protocol population			PPPDs, per-protocol population		
	Standard(N = 22)	Restricted (N = 26)	P value	Standard (N = 12)	Restricted(N = 20)	P-value
T½*—Median, [CI]^^ **Pre**operative values	40 [33–57]	45 [36–50]	0.619			
T½*—Median, (IQR^†^) [CI]^^ **Post**operative values	104 (71–370) [74–369]	159 (57–346) [61–204]	0.893	193 (74–372) [77–372]	165 (56–361) [61–274]	0.558
Delayed gastric emptying by scintigraphic criteria—No. (%) [Confidence interval]^§^	10 (45) [27–65]	13 (50) [32–68]	0.779	7 (58) [32–81]	11 (55) [34–74]	0.854
% RA 120^‡^—Median, (IQR) [CI]^^	47 (28–95) [30–94]	64 (25–96) [35–91]	0.852	84 (25–99) [40–95]	80 (24–100) [35–95]	0.906

* T½, time in minutes needed to empty half of the gastric content.

^†^ IQR, inter quartile range.

^^^^CI, bootstrap based bias-corrected 95% confidence interval;

^§^ Confidence intervals calculated using by the modified Wald method: p’ = S+2/n+4 W = 2√p’(1-p’)/n+4; CI = p’- W to p’+W. Graphpad software.

^‡^ % RA 120, percentage of radioactivity remaining in the stomach after 120 minutes of emptying.

When analyzing only those patients who underwent a PD, the median T½ was 193 minutes in the S group (95% confidence interval: 77–372) and 165 minutes in the R group (95% confidence interval: 61–274) (*P* = 0.558, NS). In the S group, 7 patients (58%) had delayed gastric emptying according to consensus recommended scintigraphy criteria, compared to 11 (55%) in the R group (*P* = 0.854, NS). The median % RA 120 was 84% in the S group (95% confidence interval: 40–95) and 80% in the R group (95% confidence interval: 35–95) (*P* = 0.906, NS). The primary outcome parameter (gastric emptying time) did not show a statistically significant difference between groups.

### Delayed gastric emptying

The incidence of delayed gastric emptying (DGE) according to the criteria of the ISGPS is shown in Table 3. There were no significant differences between both groups in the incidence of the DGE of any grade, the distribution among the different grades or the incidence of clinically relevant DGE (grade B or C).

**Table 3:** Distribution of clinical delayed gastric emptying of 54 patients who underwent PPPD* or palliative bypass procedure with GJ^†^ and were randomized between a Standard or a Restricted intraoperative fluid protocol.

	Standard (N = 24)	Restricted (N = 30)	P-value
ISGPS^‡^ grade No. (%)			
**No DGE** ^§^	12 (50) [31.4–68.6]	17 (57) [39.2–72.7]	0.524
**Grade A**	2 (8) [1.2–27]	3 (10) [2.7–26.4]	
**Grade B**	7 (29) [14.7–49.4]	4 (13) [4.7–30.3]	
**Grade C**	3 (13) [3.5–31.8]	6 (20) [9.1–37.7]	

*PPPD, Pylorus-preserving pancreatoduodenectomy;

^†^GJ, gastrojejunostomy;

^‡^ ISGPS, International Study Group of Pancreatic Surgery.

^§^ DGE, delayed gastric emptying. Delayed gastric emptying Grade A, B and C in increasing severity, (Surgery 2007; 142(5):761–768). Values shown as N, (%) and [95% confidence interval]. Confidence intervals were calculated using by the modified Wald method: p’ = S+2/n+4 W = 2√p’(1-p’)/n+4; CI = p’- W to p’+W. Graphpad software.

### Other complications

Complications other than DGE are shown for 66 patients in Table 4. Overall morbidity rates in the S and R groups were 47% and 62%, respectively (*P =* 0.32). There were no significant differences between the groups in surgical complications, relaparotomy rate or mortality. Non-surgical complications occurred in 3 patients (9%) in the S group, and in 8 patients (24%) in the R group. Cardiopulmonary complications occurred more often in the R group as compared to the S group (5 (15%) versus 1 (3%). However, this difference was not significant (*P* = 0.20). In the R group, three patients had pneumonia, there was one case of pulmonary embolism and one case of cardiac failure. In the S-group, one patient had a cardiac arrest.

**Table 4:** Perioperative outcomes of 66 patients randomized between a Standard or Restricted intraoperative fluid protocol, who underwent PD* or palliative bypass procedure with GJ^†^.

	Standard (N = 32)		Restricted (N = 34)		P-value
**Any complication—No. (%) [CI]** ^~^	15	(47) [31–64]	21	(62) [45–76]	0.32
**Surgical** complications No. (%) [CI]					
Any surgical complication	15	(47) [31–64]	20	(59) [42–74]	0.46
Pancreatic fistula^‡^	4	(13) [4–29]	8	(24) [12–40]	0.34
Postpancreatectomy hemorrhage^‡^	3	(9) [2–25]	2	(6) [1–20]	0.67
Hepaticojejunostomy leakage	3	(9) [2–25]	1	(3) [0–16]	0.35
Wound infection	5	(16) [6–32]	7	(21) [10–37]	0.75
Other	2	(6) [1–21]	5	(15) [6–31]	0.43
**Non-surgical** complications No.(%) [CI]					
Any non-surgical complication	3	(9) [2–25]	8	(24) [12–40]	0.19
Cardiopulmonary	1	(3) [0–17]	5	(15) [6–31]	0.20
Urogenital	2	(6) [1–21]	3	(9) [2–24]	1
Other	2	(6) [1–21]	5	(15) [6–31]	0.43
**Other outcomes**					
Relaparotomy—No. (%)	2	(6) [1–21]	2	(6) [1–20]	1
Hospital mortality—No. (%)	1	(3) [0–7]	1	(3) [0–16]	1
Length of hospital stay in days—Median [CI]^#^ ^§^	10	[9–17]	12	[11–14]	0.58

*PD, pancreatoduodenectomy;

^†^GJ, gastrojejunostomy; Fisher exact test.

^~^ [CI],Confidence intervals calculated by the modified Wald method: p’ = S+2/n+4 W = 2√p’(1-p’)/n+4; CI = p’- W to p’+W. Graphpad software.

^‡^ Grade B or C according to the International Study Group of Pancreatic Surgery classification;

^#^ N: 31 versus 33,

^§^Bootstrap based bias-corrected 95% confidence interval, Kruskal-Wallis test.

### Follow-up

**Fig 3:**
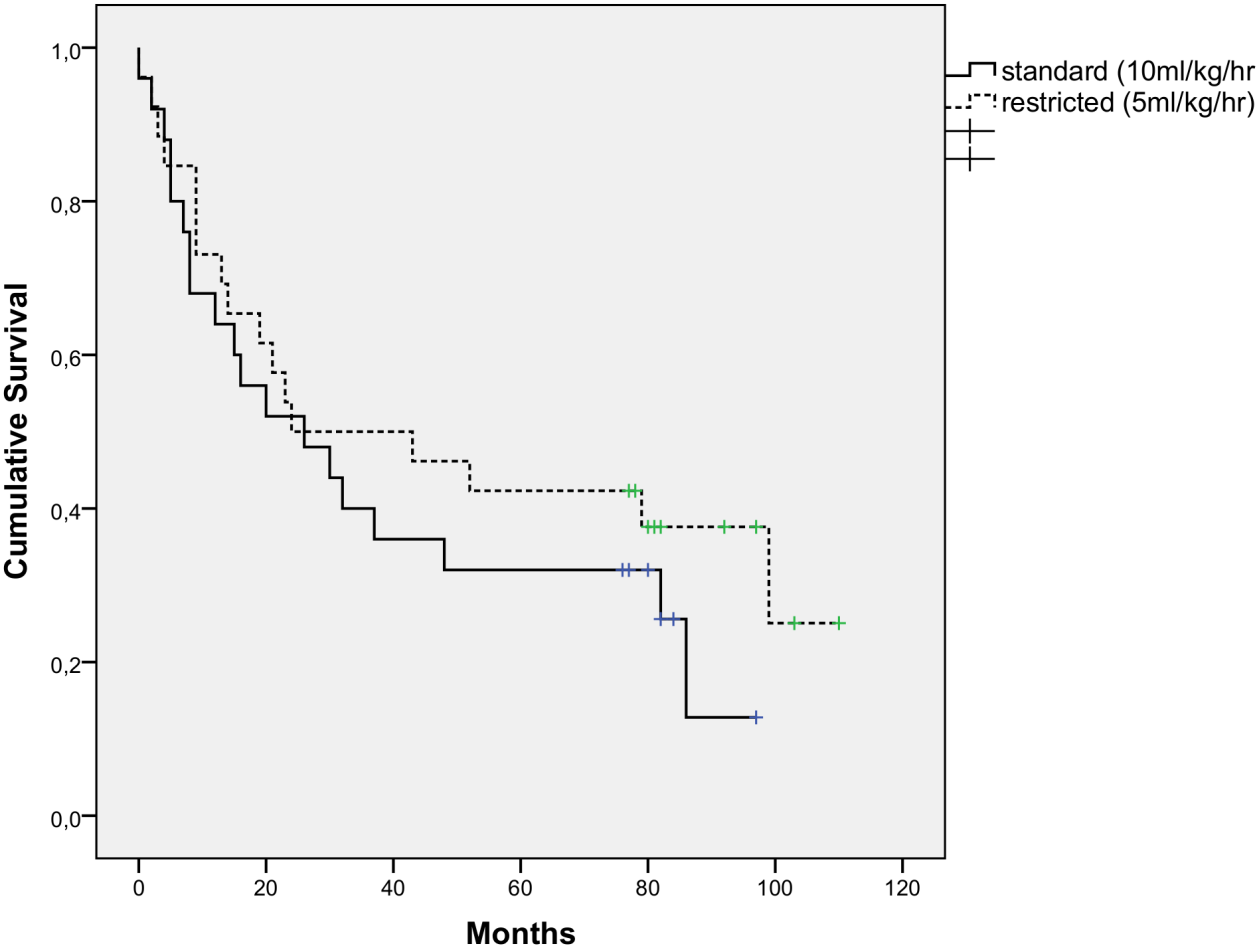
Kaplan Meier curve of cumulative survival. 6-year follow-up after operation, 66 patients randomized between a Standard or Restricted intraoperative fluid protocol. Data of 15 patients are not included (unknown status). Date of death unknown for 2 patients in standard group.

We had planned follow-up for at least a year. We recently verified the status of all randomized patients, to check for survival and possible differences between groups. This was done in August 2015, and offers a six year follow-up concerning survival. (Table 5, Fig 3)

**Table 5:** Six year follow-up regarding survival in of 66 patients randomized between a Standard or Restricted intraoperative fluid protocol, who underwent PD* or palliative bypass procedure with GJ^†^.

	Standard (N = 32)	Restricted (N = 34)	Total N	P value
Died No (%) [CI]^~^	19 (59) [42–74]	17 (50) [34–66]	36	0.47
Alive No (%) [CI]^~^	6 (19) [9–36]	9 (26) [14–43]	15	0.56
Unknown No (%)[CI]^~^	7 (22) [11–39]	8 (24) [12–40]	15	1
Total	32	34	66	

*PD, pancreatoduodenectomy;

^†^GJ, gastrojejunostomy;

^~^ [CI],Confidence intervals calculated by the modified Wald method: p’ = S+2/n+4 W = 2√p’(1-p’)/n+4; CI = p’- W to p’+W. Graphpad software.

## Discussion

This study was performed to investigate the effect of intraoperative crystalloid fluid restriction on postoperative gastric emptying in patients undergoing pancreatic surgery. Our findings were that implementation of a restricted crystalloid fluid protocol during surgery of 5 ml/kg/hr did not affect gastric emptying time on postoperative day (POD) 7, compared to the standard crystalloid fluid implementation of 10 ml/kg/hr. No differences in secondary outcome parameters, such as other complications, fistula rate, relaparotomy rate or mortality, were found. The latter results have to be considered explorative, as the sample size refers to gastric emptying. The sample size was based on an assumption which later proved to be incorrect: we assumed a normally distributed normal gastric emptying of 60 minutes with a common standard deviation of 33 minutes (between groups) based on earlier (volunteer) studies, but our preoperative gastric emptying scans showed a not normal distribution, and our median postoperative gastric emptying times had a very wide 95% confidence interval. Even if the difference in median gastric emptying times between groups was higher than 30 minutes, the sample size was not sufficient because of these results.

Randomization and blinding: our randomization was done using a computer generated list by the epidemiologist, with proper documentation and double checking of records by two independent researchers. In addition, the implementation of blinding during the operation, followed by blinding until the end of the study, led to heated discussions when in doubt for safety of the patients: for instance, when patients do not produce diuresis postoperatively (even temporarily), or for that matter, show any problem whatsoever, it is very difficult to convince the attending physician to adhere to the protocols of the trial. For this reason, we monitored blood levels of urea and creatinine, but found no significant differences between groups. Further, we should have only included the actual pancreatoduodenectomy patients. That would have created a more homogenous group. Without blinding, we would have had more knowledge about the distribution of patients between the two groups.

Various studies have shown a positive effect of crystalloid fluid restriction. However, not all studies demonstrate this positive effect, and warrant caution [20, 30]. Conflicting results of studies on fluid restriction could be explained by a lack of consensus about restricted fluid protocols, the use of fast track protocols and by the varying endpoints that were studied [12, 13, 30, 31]. The definition of restriction, when it should be applied, and which fluids must be used are still under debate. In the present study, we chose to individualize the pragmatic restricted fluid protocol based on the patient’s body weight. The primary endpoint, gastric emptying time, was based on the findings of the above-mentioned studies, which found a beneficial effect of restriction on the recovery of gastrointestinal function [3]. We chose this endpoint for several reasons. At first, to show a significant influence on a parameter with low incidence such as mortality (3% in our hospital), we would have to include an enormous amount of patients. Secondly, a substantial part of perioperative morbidity, such as pancreatic fistula, abdominal fluid collection, hemorrhage, and anastomotic leakage, directly or indirectly leads to gastric emptying problems.

We did not find beneficial effects of fluid restriction on gastric emptying, neither clinically nor in scintigraphy studies. An explanation is that fluid restriction was limited to the intra-operative phase, and that the difference in fluids was too small to result in differences in outcome (during PPPD: S group 3.4 liters vs. R group 2.1 liters, Table 1). Randomization in combination with unexpected palliative procedures had led to overrepresentation of pylorus preserving pancreatoduodenectomy (PPPD) in the restricted fluid group (23 restricted vs 13 standard). The PPPD has a much longer operation time than the palliative procedures. When these patients receive less fluid, the total amount of fluid approximates that of a palliative procedure which is twice as short but gets twice the amount of fluid, if allotted to the standard group. That is the reason why a greater difference is absent between the actual amounts of fluid given in the two groups, during the operation. MacKay and co-authors also reported no differences in outcome between two different intraoperative fluid regimens [4]. We arbitrarily chose 5 and 10 ml/kg/hr for this patient group, based on our current practice and information from earlier studies [24]. Fluid load and morbidity risk has the shape of a U-curve, meaning that risk is lowest in the middle [25]. We would probably have found differences if the fluid regimens were more extreme, but at the cost of patient safety.

Our study has some limitations. The assessment of 200 potential patients resulted in 66 randomized patients, of which 54 could finally be included in analysis of results in Table 1. Furthermore, the sample size is relatively low reflecting the difficulty to perform such a randomized trial. The exclusion of patients participating in other trials also is a limitation of this study. For the primary endpoint (gastric emptying), a sample size calculation was performed assuming a normal distribution. However, the measured gastric emptying times were not normally distributed, making the difference of 55 minutes between groups not significant. Thus, we are not yet convinced that a restricted intraoperative fluid protocol has positive effects (on gastric emptying time) in these patients. Secondly, the proportion of patients undergoing PD or palliation with GJ differed between the two study groups: 54% of patients underwent PD in the S group, versus 77% in the R group. This may have influenced the results based on selection, and certainly makes interpretation more difficult. However, in our additional, tentative subgroup analysis of patients undergoing PD, we also did not observe differences in gastric emptying time.

Our study is the first to investigate the effect of intraoperative fluid restriction in this particular patient group, in a randomized, double blinded fashion. The fluids were administered according to weight of the patients, without the use of transesophageal Doppler or monitoring of stroke volume variation, in a design resembling common practice in most hospitals. As there are no randomized controlled trials including the entire intra- and postoperative period, which demonstrate superiority of goal directed infusion therapy over plain restriction, we believed this to be the best approach at the time the trial was conducted [32–34]. Furthermore, in a recent study, goal directed therapy was of no value within an enhanced recovery protocol incorporating fluid restriction [35]. Nevertheless, this is a limitation of this study.

The primary outcome measure was determined by gastric emptying scintigraphy, which is an objective, validated method to assess gastric emptying time even in the postoperative period of pancreatic surgery. The homogenous population of patients all planned to undergo the same procedure, makes results specific to this group.

## Conclusion

In the present study, no significant differences in (delayed) gastric emptying or other complications were found between standard or restrictive fluid management. However, a larger study would be needed to draw definite conclusions about fluid restriction in pancreatic surgery.

## Supporting Information

S1 CONSORT Checklist Consort 2010 Checklist.doc. (DOC)

S1 File Ethical committee AMC.pdf. Letter from ethical committee pertaining to inclusion of extra patients.
(PDF)

S2 File Syntax1 tablesBCAcorr1408.sps. Original syntax used to analyse database, resulting in tables.
(SPS)

S1 Protocol Whipple protocol vs.5.6 april 24.doc. Original trial protocol.
(DOC)
