# Supplementary material for: Intraoperative Fluid Restriction in Pancreatic Surgery: A Double Blinded Randomised Controlled Trial
Source: PLoS One. 2015 Oct 14;10(10):e0140294. doi: 10.1371/journal.pone.0140294 (PMC4605599; doi:10.1371/journal.pone.0140294)
Supplement: S1 File — Letter from ethical committee pertaining to inclusion of extra patients. (PDF) [file pone.0140294.s002.pdf]

Academisch Medisch Centrum
Universiteit van Amsterdam

Prof.dr. M.W. Hollmann
Anesthesiologie
H1-112

Medisch Ethische Commissie
E2-236
doorkiesnummer: 566 7389/566 5880
fax: 5669015

Amsterdam, 20 april 2009
uw kenmerk:
ons kenmerk: MEC 04/160 # 09.17.0629
betreft:
MEC 04/160 uw project:
A randomised controlled trial on gastric motility effects induced by fluid restriction in patients undergoing elective pancreatic surgery. EPOR-trial.

Geachte heer Hollmann,

Hiermee bevestigen wij de ontvangst van uw brief d.d. 24 maart 2009 betreffende het aantal in bovengenoemde studie geïncludeerde patiënten.

Wij delen u gaarne mee dat wij deze rapportage voor kennisgeving hebben aangenomen.

Met vriendelijke groet,
namens de Medisch Ethische Commissie,

Mw.mr. M.L.M. van der Hulst,
secretaris

Beste Jan,
Denk ook nog om aanmelden studie bij trial buro.
Groet Jan

amc

Academisch Medisch Centrum

Universiteit van Amsterdam

Prof.dr. M.W. Hollmann
Anesthesiologie
H1-112

Medisch Ethische Commissie
E2-162
doorkiesnummer: 566 5880
fax: 5669015

Amsterdam, 12 januari 2006
uw kenmerk:
ons kenmerk: MEC 04/160 # 06.17.0120
betreft:
MEC 04/160 uw project:
A randomised controlled trial on gastric motility effects induced by fluid restriction in patients undergoing elective pancreatic surgery. EPOR-trial.

Geachte heer Hollmann,

In vervolg op de ontvangst van uw reactie d.d. 9 januari 2006 op onze brief van 4 januari jl. delen wij u inzake bovengenoemd project, ons ter beoordeling voorgelegd op 15 juni 2004, gaarne mee dat onze commissie

- tot oordelen bevoegd krachtens artikel 2, tweede lid, onder a, van de Wet medisch-wetenschappelijk onderzoek met mensen (WMO);
- werkzaam volgens de ICH-GCP richtlijnen;
- op grond van de haar voorgelegde stukken als hierna vermeld;
- gelet op artikel 3 van de WMO;
- vastgesteld hebbend dat aan de beoogde proefpersonen op adequate wijze informatie wordt gegeven over het uit te voeren onderzoek;
- vastgesteld hebbend dat voor het onderzoek een verzekering is gesloten conform de WMO en het Besluit verplichte verzekering bij medisch-wetenschappelijk onderzoek met mensen,

heeft besloten tot een positief oordeel over deze studie en de uitvoering daarvan in het AMC.

In de beoordeling betrokken documenten:

- protocol versie 5.4 d.d. 9 januari 2006;
- patiënteninformatie/toestemmingsverklaring versie 2.4 d.d. 9 januari 2006;
- advies Adviescommissie Stralenbelasting d.d. 16 november 2005;
- ABR-formulier;
- AMC-appendix;
- protocolsamenvatting.

Wij verzoeken u onze commissie op de hoogte te stellen van de daadwerkelijke start van het onderzoek, van de (al dan niet voortijdige) beëindiging daarvan, en van tijdens de studie optredende onverwachte complicaties. Voorts dienen eventuele protocolwijzigingen ter beoordeling aan onze commissie voorgelegd te worden.

Wij wijzen u erop dat op grond van artikel 23 van de Wet medisch-wetenschappelijk onderzoek met mensen degene wiens belang rechtstreeks bij een besluit van de MEC is betrokken, daartegen binnen zes weken na de dag waarop het besluit bekend is gemaakt, een administratief beroepschrift kan indienen bij de Centrale Commissie Mensgebonden Onderzoek. Een dergelijk administratief beroepschrift dient geadresseerd te worden aan: CCMO, Postbus 16302, 2500 BH Den Haag.

Ten tijde van de beoordeling van dit project was de commissie als volgt samengesteld:

prof. dr. J.D. Bos	:	voorzitter, hoogleraar dermatologie
prof.dr. P.M.M. Bossuyt	:	plv. lid, hoogleraar klinische epidemiologie
dr. H.H.F. Derkx	:	kindergastro-enteroloog
dr. A. Hijdra	:	neuroloog
mw. mr. M.L.M. van der Hulst	:	secretaris, jurist
mw. L. Kok-Noorman	:	beoordeelt onderzoek specifiek vanuit de invalshoek van de patiënt

Meibergdreef 9 Postbus 22660 1100 DD Amsterdam Telefoon (020) 566 9111 Fax (020) 566 4440

Academisch Medisch Centrum

Universiteit van Amsterdam

mw.dr. N.E. Langeveld	:	onderzoeksverpleegkundige
dr. J.F.M. Slors	:	chirurg
prof. dr. J.G.P. Tijssen	:	hoogleraar klinische epidemiologie van hart- en vaatziekten
mw.dr. M.D. Trip	:	internist
drs. A. Vyth	:	ziekenhuisapotheker
mw.dr. A.M. Westermann	:	internist-oncoloog
prof.dr. D.L. Willems	:	hoogleraar medische ethiek.

Met vriendelijke groet,
namens de Medisch Ethische Commissie,

b.a. Tijssen

Prof.dr. J.D. Bos,
voorzitter

Mw.mr. M.L.M. van der Hulst,
secretaris

c.c. CCMO
c.c. AMC Medical Research B.V. (+)

1141841
