# Supplementary material for: Intraoperative Fluid Restriction in Pancreatic Surgery: A Double Blinded Randomised Controlled Trial
Source: PLoS One. 2015 Oct 14;10(10):e0140294. doi: 10.1371/journal.pone.0140294 (PMC4605599; doi:10.1371/journal.pone.0140294)
Supplement: S1 Protocol — Original trial protocol. (DOC) [file pone.0140294.s004.doc]

**PROTOCOL FOR A CLINICAL TRIAL EPOR-trial**

Effects of Per-Operative fluid Restriction in patients undergoing pancreatic surgery

## 1 Full study title

A Randomised controlled trial on gastric motility effects induced by fluid restriction in patients undergoing elective pancreatic surgery

# 2 Investigators

**2.1 Principal investigator**

Prof.dr. M.W. Hollmann, anaesthesiologist-molecular biologist

Dep. of Anaesthesiology AMC/UoA

**2.2 Senior investigators**

Prof.dr. D.J. Gouma, surgeon Dep. of Surgery AMC/UoA

**2.3 Investigators**

G. van Samkar, anaesthesiologist; Dep. of Anaesthesiology AMC/UoA

Dr. O.R.C. Busch, surgeon; Dep. of Surgery AMC/UoA

Dr. R. Bennink Dep of Nuclear Medicine AMC/UoA

Dr. J. Hofland, anesthesiologist-intensivist Dep. of Anaesthesiology AMC/UoA

**2.4 Statistics**

M. G. Dijkgraaf, statistician Dep. of Biostatistics AMC/UoA

3 Abbreviations and / or definitions:

| ASA | American Society of Anesthesiologists |
| --- | --- |
| BW | Body Weight (kg) |
| Mean | Arithmetic mean |
| GCP | Good Clinical Practice |
| NIBP | Non-Invasively derived Blood Pressure (mmHg) |
| AP | Arterial Pressure (mmHg) |
| HR | Heart Rate (beats/min) |
| i.v. | Intra-venously |
| FiO_2_ | Inspiratory oxygen portion (%) |
| PaO_2_ | Partial pressure of arterial oxygen tension (kPa) |
| PaCO_2_ | Partial pressure of arterial carbon dioxide tension (kPa) |
| IPPV | Intermittant Positive Pressure Ventilation |
| PEEP | Positive End-Expiratory Pressure (cm H_2_O) |
| CRF | Case Record Form |
| DGE | Delayed Gastric Emptying: a nasogastric tube is still necessary at/after the 10^th^ post-operative day and/or there is no solid food intake at/after the 14^th^ post-operative day |
| SRC | Surgery Related Complications |
| GC | General Complications |
| CAL | Caloric intake: amount of oral food intake by the patient (Kcal (KJ)/day) |
| BGA | Blood Gas Analysis |

**4 Background**

Surgery induces tissue trauma with losses of body fluids and blood as a consequence. Usually these losses are replenished during the operative and early post-operative periods. However, the combination of tissue trauma and fluid replenishment may be responsible for the formation of oedema, e.g. at the gastro-intestinal tract. Recently published studies reported a beneficial influence on the recovery of the gastrointestinal function in humans and a reduction in post-operative complications when the amount of fluid given in the peri-operative period was restricted.^1,2,3^ Nonetheless, recent reviews still call for controlled trials comparing dry and wet regimes in well defined groups^4,5^.

Delayed gastric emptying (DGE) often occurs after performing pancreatic surgery, in particular after pancreatico-duodenectomy.^6-9^ Various causes and therapies in relation to this problem were studied as were the outcome and complication rate of this operation using standard methods of treatment.^10-12,13-24^ DGE is influenced by many factors, and is related to postoperative complications.^25,26^ A recent study ^1^ demonstrated significant reduction of solid and liquid phase gastric emptying time in patients after elective colonic resection, in the group with postoperative restriction of salt and water intake.. However, there was no standardisation of intravenous fluid regime during the operation in this study. Our hypothesis is that peroperative fluid restriction will lead to a significant reduction of solid phase gastric emptying time – measured by radionuclide scintigraphy-, and a reduction in its related postoperative complications.

### 5 Trial Objective

**5.1 Trial Objective**

The aim of this study is to evaluate possible effects of a restricted per-operative fluid infusion regime on solid phase gastric emptying time in patients undergoing a pancreatico-duodenectomy in comparison with an accepted and standardised fluid infusion regime.

**5.2 Study Population**

Eligible patients for participation in this clinical trial are those planned to undergo elective pancreatico-duodenectomy regardless the use of a pylorus-preserving technique. Patients are only allowed to participate in this study after they have given their written informed consent and when meeting all the inclusion criteria and not exhibiting any exclusion criterion.

## 6 Trial Design

The study is designed as a prospective, double blinded, single centre randomised controlled trial. The total number of patients enrolled into this trial will be 44 (see 11.2 for power calculation).

**6.1 Primary and Secondary End-points**

The *primary endpoint* is defined as the reduction of minutes needed to achieve a 50% emptying of the stomach (T50) due to a restricted fluid infusion regime. On the 1^st^ preoperative day, a baseline T50 will be established using a radio-labelled pancake, containing 12 MBq ^99m^Tc-colloid. This will be measured again on the 7^th^ postoperative day, using radionuclide scintigraphy.

The *secondary endpoints* are the following:

- Total amount used of: furosemide, (no prior usage)
- Total amount used intra-operatively of: noradrenalin.
- Blood Urea and creatinine levels: a rise of more than 10% of pre-operative values measured at: pre-assessment vs. day 1, 3 and 7 postoperatively.
- Albumin levels: day 1, 3 and 7 postoperatively.
- Nutritional intake (calculation by dietician)
- Duration of hospital stay
- The length of remaining duodenum will be measured during operation (distance between pylorus and duodeno-jejunostomy)

All complications will be recorded in the CRF.

6.1a **Method of gastric emptying measurement:**

1. Patients must have fasted at least 4 hours before start of ingestion of the radio-labelled pancake.

2. The pancake must be ingested for at least 75% before the scintigraphy starts.

3. Patients not capable of ingesting the pancake within 5 minutes will be excluded from the delayed gastric emptying analysis.

4. Patients still having a nasogastric drainage tube on the 7^th^ postoperative day will not be excluded from this gastric emptying measurement technique, and thus will follow the same examination as patients without having a nasogastric tube on the 7^th^ postoperative day.

5. If a patient vomits after ingestion of the pancake during or before the scintigraphy, the gastric emptying is assumed to be 0%. If no gastric emptying is measured, the *amount* of gastric emptying is set as 0%.

6. Prokinetics which may influence the gastric emptying, - meaning Domperidon (Motilium^R^), and Metoclopramide (Primperan^R^)- will not be used on the day of gastric emptying scintigraphy in the study population,.

7. 5-HT3 receptor antagonists such as Ondansetron (Zofran^R^) may not be used 24 hrs prior to the gastric emptying examination. This is also applicable to other antiemetics.

8. Erythromycin is not to be used in the days before the gastric emptying scintigraphy.

**6.1b Calculation and evaluation of the nutritional intake:**

**1. Preoperative**:

On the preoperative day the dietician will instruct the patient how to fill in a diary postoperatively. In this diary the patient will record his oral intake (food and fluids) with as much detail as possible. Nurses will help the patient to complete the diary in the event that the patient is not capable of recording his intake.

The dietician will check and calculate the oral intake on a daily basis.

*Baseline measurements*:

Weight (kg)

Height (m)

Body Mass Index (calculated by the dietician).

**2. Postoperative introduction of fluids and food:**

- Postoperatively, the total amount of fluids (including intravenous fluids) will be maximized to 2.5 litres per 24 hours. The iv. Fluid will be: Ringers Lactate. The calculation is on the basis of 30 ml/kg body weight, and will be set to the value nearest to 2000ml or 2250ml or 2500ml.
- The nasogastric tube will be removed when it produces 300 ml or less per 24 hours.
- After the removal of the nasogastric tube the patient will start to expand oral intake under supervision of the dietician.

Nutrition builds up day by day, if possible:

Day 2 postoperative: fluid (V2)

Day 3 postoperative: fluid extended (V3)

Day 4 postoperative: solid food (GD)

Administration of intravenous fluids is decreased with successful increase of oral intake

After the introduction of oral nutrients, the patient starts the registration of the daily oral intake

Postoperative weight control, if possible, on:

Day 2

Day 5

Day 8

Day 11

Day 14

**2. Evaluation of the nutritional intake**:

Definition of optimal nutrition during metabolic stress:

1.5 grams of protein per kilogram bodyweight

30 kcal per kilogram bodyweight

30 ml oral fluid per kilogram bodyweight

measurement of N- balance (using urea concentration in urine)

**Goal of the nutritional study**: to reach 50% of the nutritional protein target for at least 2 consecutive days. To evaluate possible relapses, the patient is asked to continue the registration of the nutritional intake and the dietician will continue to calculate the patients’ intake.

**6.2 Anaesthesia**

Patients will be premedicated with lorazepam. In the operating room, usual anaesthetic monitoring will be started. After placement of a thoracic epidural catheter a bolus (bupivacaine 0.25% with adrenaline 1:100,000; 3 ml) will be used as testing dose for preventing accidental placement of the epidural catheter into the spinal space. I.v. induction of anesthesia will be with Sufentanil (0,2 to 0,4 mcg/kg BW), etomidate (0.2-0.3 mg/kg BW) and Rocuronium (0.5 mg/kg BW). After tracheal intubation, the lungs will be ventilated using IPPV with settings of FiO_2_ 0.60 (oxygen-air mixture), frequency 12 breaths/min, tidal volume 7 ml/kg BW, PEEP 5 cm H_2_O, and I/E ratio 1:1.5. Respiratory frequency or volume will be adjusted to maintain PaCO_2_ between 4.4 and 6.3 kPa. Set PEEP and I/E ratio will not be changed during the procedure. FiO_2_ can be changed if hypoxia occurs (PaO_2_ < 10 kPa). A nasogastric tube will be inserted and anaesthesia will be maintained with isoflurane (0.8 % end tidal), Rocuronium (10mg bolus,will be used if clinically necessary). A top-up dose injected via the epidural catheter of bupivacaine 0.25%,(5ml =12.5 mg) and Sufentanil 25 mcg (5ml) – total volume being 10ml - given 10 min before the start of the surgical procedure. If analgesia is considered insufficient, extra i.v. Sufentanil will be administered. Two hours after the surgical procedure has begun the patients will start with a continuous infusion via the epidural catheter with bupivacaine 0.25% and Sufentanil 0,45 mcg/ml (infusion rate 8- 10 ml/h) using an infusion pump. This epidural infusion will be continued for three days postoperatively till the catheter is removed during the post-operative period on the fourth postoperative day. Postoperative analgesia is tested daily, using cold-warm discrimination and pinprick test, as well as a VAS score to determine patient levels of analgesia. At the end of the operation, the neuromuscular blockade will be antagonized if necessary. In all patients arterial blood pressure will be measured. (Arterial cannulation of the left radial artery is preferred)

For fluid management the distribution of the patients follows the randomisation schedule, separating the following groups:

**Group S (Standard):**

1. Maintenance always with i.v. Ringer’s lactate:

1. First hour: 10 ml/kg BW
2. After the first hour: 10 ml/kg BW/h

2. For management of blood loss:

1. Initially 500 ml Hes 130 / 0,42 (Venofundin®) i.v. for expected blood loss.
2. If blood loss < 500 ml: second Hes 130 / 0,42 (Venofundin®), 500 ml i.v. (volume to volume, so all blood loss is replaced by Hes 130 / 0,42 (Venofundin®)
3. If blood loss <1,000 ml: third Hes 130 / 0,42 (Venofundin®), 500 ml i.v.
4. If blood loss < 1,500 ml: i.v. infusion of packed red cell (PC), guided by haemoglobin (Hb) level

- Patient < 70 years: infusion trigger Hb = 5.0 mmol/l
- Patient ≥ 70 years: infusion trigger Hb = 5.5 mmol/l

1. If the first i.v. infusion of Hes 130 / 0,42 (Venofundin®) is ≥ 6 hours before, a fourth may be given, otherwise the infusion of 2 units PC’s will be alternated with 1 unit ESDEP.

3. If mean AP is >20% below baseline (see 8.2) then i.v. noradrenalin (standard dose: 1-10 mcg/kg/h) will be given for correction. Mean AP must be kept in the range of 20% +/- baseline.

4. Post-operative fluid management will be done with i.v. Ringers Lactate, 2.5 l/24h.

Group R (restricted)

1. Maintenance always with i.v. Ringer’s lactate:

1. First hour: 5 ml/kg BW plus Hes 130 / 0,42 (Venofundin®), 500 ml
2. After the first hour: 5 ml/kg BW/h

2. For management of blood loss:

1. No initial infusion for expected blood loss.
2. If blood loss < 500 ml; then replacement with second Hes 130 / 0,42 (Venofundin®), 500 ml (volume to volume, so all blood loss is replaced by Hes 130 / 0,42 (Venofundin®)
3. If blood loss < 1,000 ml; then replacement with third Hes 130 / 0,42 (Venofundin®), 500 ml
4. If blood loss < 1,500 ml; then replacement with fourth Hes 130 / 0,42 (Venofundin®), 500 ml unless haemoglobin level infusion triggerpoints are passed, then replacement will be with i.v. infusion of Packed red Cell (PC)

- Patient < 70 years: infusion trigger Hb = 5.0 mmol/l
- Patient ≥ 70 years: infusion trigger Hb = 5.5 mmol/l

e. The infusion of 2 units PC’s will be alternated with 1 unit ESDEP.

3. If mean AP is >20% below the baseline (see 8.2) then i.v. noradrenaline (standard dose 1-10 mcg/kg/h) will be given for correction. Mean AP must be kept in the range of 20% +/- baseline.

4. Post-operative fluid management will be done with i.v. Ringer’s Lactate, 2.5 l/24h

During the course of the study, procedures will be carried out according to the time schedule shown at appendix 2 (also see CRF)

**6.3 Measures to avoid /minimize bias**

**6.3.1 Randomization**

Patients will be randomized using a computer generated randomization procedure. Patients will be randomly assigned to receive either a “**Standard**” or a “**Restricted**” fluid regime.

**6.3.2 Blinding**

The attending anaesthesiological and surgical team as well as the patient are blinded as to the regime followed. One of the investigators will set the fluid regime at the start of the procedure according to the randomisation (group R or S) the patient was allocated to. The set regime then will be hidden for the attending medical team (e.g. by using a clothes bag). Thus, the attending medical team members are unable to control the fluid regime during the operation, and do not know to which group the specific patient was allocated to. The postoperative care is standardised in such a way that there will be no treatment difference between the groups.

**6.4 Duration of study participation**

The participation duration per patient will be until discharge from the hospital. In case of persistent adverse events and/or intercurrent diseases, appropriate medical measures have to be taken; in such circumstances the patients have to be closely monitored until the problems are solved.

**6.5 Stopping rules or discontinuation**

A patient may be withdrawn from the study for the following reasons:

- Withdrawal of consent
- Not cooperative, non compliance
- Failed epidural analgesia, meaning conversion to PCA or continuous morphine infusion postoperatively.
- Any reason which, in the eyes of the investigator, does not justify continuation of the study
- Serious adverse events / intra-operative events : myocardial infarction, admission to intensive care after operation, peroperative blood loss of > 50% of circulating volume (usually set at 70 ml/kg BW for an adult)

## 7 Selection and Withdrawal of subjects

Prior to possible recruitment, all patients will be thoroughly examined. They will undergo detailed physical examination (screening) including the following:

- Medical History
- Age, weight, height, ethnic origin, sex.
- Previous anaesthesia
- Concomitant diseases
- Concomitant medication
- ECG (12-lead)
- Body weight
- Non invasively derived Blood pressure.
- Laboratory tests (including level of Haemoglobin, and plasmalevels of sodium, potassium, urea and creatinine,)

**7.1 Subject inclusion criteria**

The patients must comply with the following criteria in order to be eligible to participate in this clinical study:

- Age range ≥ 18 years
- Male patients, or female patients of non childbearing potential or with adequate contraception
- ASA classification I – IV
- Patients who will undergo elective pancreatic surgery
- Written informed consent

All inclusion criteria must be met; otherwise the patient cannot be enrolled into the study.

**7.2 Subject exclusion criteria**

During the screening period, it must be demonstrated that the following criteria do not apply:

- Age range: < 18 years
- ASA classification V
- Emergency operations
- Pregnancy
- Breast feeding period
- Informed consent missing
- Alcohol abuse (more than 35 units a week)
- Drug abuse (opiates, cocaine)
- SaO_2_ < 90% (room atmosphere) SpO_2_< 8 kPa
- Presumed non cooperatives
- Legal incapacity
- Refusal to undergo epidural anaesthesia
- Dialysis or fluid restriction based on renal failure
- Any clinical condition which does not justify study participation in the investigator’s opinion

If one or more of these criteria apply, the patient will not be enrolled into this study.

**7.3 Type and timing of the data to be collected for withdrawn subjects**

In case of withdrawn subjects the reasons for withdrawal will be accurately documented in the CRF.

## 8 Treatment

**8.1 Treatment modalities**

Lorazepam is used as pre-medication. Analgesics are not allowed in this context. The antibiotic regime: Cefuroxim 1500mg, single shot. Sandostatine 3 times daily 0.1mg. Fraxiparine will be administered according to common hospital regime. Anaesthesia, mechanical ventilation and fluid administration are described in detail at paragraph 6.2.

**8.2 Concomitant medications**

If necessary, Rocuronium will be administered for muscle relaxation. At the end of the anaesthesia the patient will receive neostigmine, if necessary.

Mean intra-operative arterial pressure is set at the preoperatively measured non-invasively derived blood pressure (NIBP) (= baseline) +/- 20%. In order to maintain this arterial pressure, measures will be taken as decribed in section 6.2.

All perioperative medications and/or measures have to be reported and documented in the CRF.

Postoperative analgesia will consist of epidural analgesia, (bupivacaine 0.25% Astra® + Sufentanil 0,25 μg/ ml, pump rate of 8- 12 ml/h), together with paracetamol (1 g; 3-4 times per day).

**9 Adverse events**

**9.1 Definition of Adverse Events/Effects**

Adverse events are defined as symptoms or events reported spontaneously by the patient or detected by the investigator and occur in a patient. The necessity to document this information is independent of whether this event is related to the administration of a “**Standard**” or “**Restricted**” fluid regimen.

**9.2 Severity**

All adverse events and/or symptoms have to be rated according to severity, seriousness, and possible relation to the study.

The severity of each adverse event has to be classified as *mild, moderate, severe* or *extreme,* according to the following guidelines:

*Mild:* No influence on the daily activities of the patients, no symptomatic treatment necessary.

*Moderate:* Normally associated with a certain inconvenience or concern to the patients, may influence daily activities, optional use of simple therapeutic measures.

*Severe:* Associated with a considerable inconvenience or concern to the patients and necessitates in general medical treatment or other appropriate therapeutic measures.

*Extreme*: Interrupts the daily activities of the patients and necessitates medical treatment or other appropriate therapeutic measures.

**9.3. Recording of Adverse Events/Effects**

Any adverse event must be recorded on the adverse event form in the CRF. In this section the following data will be collected:

- Kind of adverse event
- Onset
- Duration
- Severity
- Course
- Relation to concomitant diseases/treatments
- Relation to the study fluid regimes

Special attention has to be paid to this section, as the evaluation of potential risks is of major importance in terms of the benefits gained by the patients. Besides, all medical interventions have to be specified in this sheet.

**10 Time Schedules**

After approval by the ethics committee patient enrollment should start by the first quarter of 2006.

## 11 Statistics

**11.1 Statistical analysis**

Standard and restricted fluid regimes will be compared on the basis of intention to treat and per protocol. The ‘intention to treat’ sample includes all patients who received the anaesthetics and reached at least 10 minutes after incision. The ‘per protocol’ sample consists of all patients having completed the study protocol according to schedule. Student’s T-tests for independent groups will be used for the between group comparisons. If the randomisation procedure by chance happens to result in initially heterogeneous groups with respect to age, gender and ASA-classification, covariance analysis will be applied to correct for differences at baseline.

**11.2 Sample size**

The number of patients is based on the following calculations: A sample size of 22 in each group will have 90% power to detect a difference in means of 30 assuming that the common standard deviation is 33.333 using a two group t-test with a 0.05 one-sided significance level. Taking into account a drop-out rate of 10%, the total number of patients in the study will be 50.

**12 Records**

**12.1 Entries**

All data entries have to be made directly in the CRF. Individual sets of CRF’s will be used for each patient. The investigator will be responsible for ensuring that all the questions on the CRF are answered fully. If certain data are not available, not done or not applicable the investigator will enter: “N.Av.”, “N.D.”, “N.Ap”, respectively, in the appropriate spaces.

The 24-hour clock will be used for all time entries. (00:00 – 23:59)

**12.2. Errors**

The CRF will be reviewed and evaluated for completeness and forms with errors or omissions will be returned to the investigator for correction. Changes or additions to the data must be made in the following manner: the original entry will be crossed out with a single line drawn through the error (not erased or whited out) so as to leave the original entry still legible. The correction should be entered in ink, initialled and dated by the person making the correction.

**12.3 Maintenance of records**

The investigator will keep a record of the full names and addresses of the patients and a copy of the signed informed consent form, which must be able to uniquely identify a patient, and the corresponding study number. These records are to be retained for a period of 15 years following the completion of the trial.

**13 Ethics**

**13.1 Informed consent**

A physician will inform the patients or patients relative about the nature, relevance and consequences of the study and they have to give their written informed consent to participate in this study. Additionally they will receive written study information. An example of the informed consent (in dutch) can be found in the appendix.

**13.2 Ethics committee**

The study protocol and the appropriate documents have been submitted and approved by the Ethics Committee.

**13.3 Declaration of Helsinki**

The investigators pledge to fully respect and follow the Declaration of Helsinki in the current version of Somerset West, SA (1996)^27^

**14 References**

References

1. Lobo DN, Bostock KA, Neal KR et al. Effect of salt and water balance on recovery of gastrointestinal function after elective colonic resection: a randomised controlled trial. Lancet 2002; 359:1812-1818.

2. Brandstrup B, Tonnesen H, Beier-Holgersen R et al. Effects of intravenous fluid restriction on postoperative complications: comparison of two perioperative fluid regimens: a randomized assessor-blinded multicenter trial. Ann Surg 2003; 238:641-648.

3. Nisanevich V, Felsenstein I, Almogy G et al. Effect of intraoperative fluid management on outcome after intraabdominal surgery. Anesthesiology 2005; 103:25-32.

4. Grocott MP, Mythen MG, Gan TJ. Perioperative fluid management and clinical outcomes in adults. Anesth Analg 2005; 100:1093-1106.

5. Holte K, Sharrock NE, Kehlet H. Pathophysiology and clinical implications of perioperative fluid excess. Br J Anaesth 2002; 89:622-632.

6. Closset J, Gelin M. Delayed gastric emptying after pancreatoduodenectomy. Acta Chir Belg 2003; 103:338-339.

7. Park YC, Kim SW, Jang JY et al. Factors influencing delayed gastric emptying after pylorus-preserving pancreatoduodenectomy. J Am Coll Surg 2003; 196:859-865.

8. Riediger H, Makowiec F, Schareck WD et al. Delayed gastric emptying after pylorus-preserving pancreatoduodenectomy is strongly related to other postoperative complications. J Gastrointest Surg 2003; 7:758-765.

9. Shan YS, Sy ED, Lin PW. Role of somatostatin in the prevention of pancreatic stump-related morbidity following elective pancreaticoduodenectomy in high-risk patients and elimination of surgeon-related factors: prospective, randomized, controlled trial. World J Surg 2003; 27:709-714.

10. Gouma DJ, van Geenen RC, van Gulik TM et al. Rates of complications and death after pancreaticoduodenectomy: risk factors and the impact of hospital volume. Ann Surg 2000; 232:786-795.

11. van Geenen RC, van Gulik TM, Busch OR et al. Readmissions after pancreatoduodenectomy. Br J Surg 2001; 88:1467-1471.

12. van Geenen RC, van Gulik TM, Offerhaus GJ et al. Survival after pancreaticoduodenectomy for periampullary adenocarcinoma: an update. Eur J Surg Oncol 2001; 27:549-557.

13. Kehlet H, Holte K. Review of postoperative ileus. Am J Surg 2001; 182:3S-10S.

14. Closset J, Gelin M. Delayed gastric emptying after pancreatoduodenectomy. Acta Chir Belg 2003; 103:338-339.

15. Aranha GV, Hodul PJ, Creech S et al. Zero mortality after 152 consecutive pancreaticoduodenectomies with pancreaticogastrostomy. J Am Coll Surg 2003; 197:223-231.

16. Karavias DD, Tepetes K, Vagenas K et al. Gastrografin administration in the treatment of postoperative delayed gastric emptying. Int J Clin Pract 2002; 56:173-174.

17. Yamaguchi K, Kishinaka M, Nagai E et al. Pancreatoduodenectomy for pancreatic head carcinoma with or without pylorus preservation. Hepatogastroenterology 2001; 48:1479-1485.

18. Spanknebel K, Conlon KC. Advances in the surgical management of pancreatic cancer. Cancer J 2001; 7:312-323.

19. Seiler CA, Wagner M, Sadowski C et al. Randomized prospective trial of pylorus-preserving vs. Classic duodenopancreatectomy (Whipple procedure): initial clinical results. J Gastrointest Surg 2000; 4:443-452.

20. Martignoni ME, Friess H, Sell F et al. Enteral nutrition prolongs delayed gastric emptying in patients after Whipple resection. Am J Surg 2000; 180:18-23.

21. Fabre JM, Burgel JS, Navarro F et al. Delayed gastric emptying after pancreaticoduodenectomy and pancreaticogastrostomy. Eur J Surg 1999; 165:560-565.

22. Thor PJ, Matyja A, Popiela T et al. Early effects of standard and pylorus-preserving pancreatectomy on myoelectric activity and gastric emptying. Hepatogastroenterology 1999; 46:1963-1967.

23. Berge Henegouwen MI, van Gulik TM, DeWit LT et al. Delayed gastric emptying after standard pancreaticoduodenectomy versus pylorus-preserving pancreaticoduodenectomy: an analysis of 200 consecutive patients. J Am Coll Surg 1997; 185:373-379.

24. Yeo CJ, Barry MK, Sauter PK et al. Erythromycin accelerates gastric emptying after pancreaticoduodenectomy. A prospective, randomized, placebo-controlled trial. Ann Surg 1993; 218:229-237.

25. Boivin MA, Carey MC, Levy H. Erythromycin accelerates gastric emptying in a dose-response manner in healthy subjects. Pharmacotherapy 2003; 23:5-8.

26. Berne JD, Norwood SH, McAuley CE et al. Erythromycin reduces delayed gastric emptying in critically ill trauma patients: a randomized, controlled trial. J Trauma 2002; 53:422-425.

27. World Medical Association Declaration of Helsinki. Recommendations guiding physicians in biomedical research involving human subjects. Cardiovasc Res 1997; 35:2-3.

Appendix: informed consent

***Informatie formulier*** *(versie 2.4_ 9_januari 2006)*

Het beperken van vocht tijdens de operatie van Whipple.

Geachte Mevrouw. Meneer,

U bent opgenomen vanwege een operatie aan uw alvleesklier. Met toestemming van uw behandelende artsen willen wij uw vragen om mee te doen aan een onderzoek. Het onderzoek bestaat uit het vergelijken van verschillende hoeveelheden infusievloeistof tijdens de operatie, als u in narcose bent.

Door de computer wordt u willekeurig in een van beide groepen (standaard hoeveelheid of weinig) geplaatst voor het onderzoek.

**Wie kan er meedoen aan het onderzoek?:**

In principe alle patiënten die aan hun alvleesklier worden geopereerd. Bij zwangerschap of bij geven van borstvoeding wordt dit onderzoek niet uitgevoerd. Bij vrouwelijke deelnemers in de vruchtbare leeftijd wordt eerst een zwangerschapstest gedaan.

**Achtergrond en doel van het onderzoek:**

De operatie die u zult ondergaan is een zware operatie, die een aantal uren zal duren. Meestal wordt er veel infusie vloeistof toegediend tijdens de operatie. De laatste jaren is bekend geworden dat minder vloeistof, mogelijk leidt tot gunstiger resultaten, en minder complicaties geeft. Er zijn echter weinig studies tot nu toe gedaan. Het doel van het onderzoek is om aan te tonen dat ook in ons ziekenhuis, minder vocht kan leiden tot verbetering van resultaten. Als dit uit het onderzoek komt, kunnen wij in de toekomst de patiënten beter en veiliger behandelen.

**Methode:**

De operatie en narcose verlopen voor u precies zoals de mensen die niet aan het onderzoek meedoen. Het enige verschil is dat u tijdens de narcose de standaard of minder infusievloeistof krijgt toegediend. Hier merkt u niets van. Tijdens de operatie wordt bijgehouden hoe uw bloeddruk is en hoeveel U plast, zaken die we altijd al doen voor zulke operaties. Alle maatregelen die nodig zijn om de narcose en operatie stabiel te laten verlopen worden bij u genomen net zoals bij mensen die niet aan het onderzoek meedoen. Op de afdeling zult u vocht via het infuus toegediend krijgen, net als andere patiënten die deze operatie ondergaan. Daarnaast zal voor en 7 dagen na de operatie een onderzoek uitgevoerd worden waarbij gekeken wordt naar het functioneren van de maagdarmmotoriek. Hiervoor vragen wij u op dat moment een pannenkoek te eten met een licht radioactieve merkstof. De totale hoeveelheid straling die hierbij vrijkomt is vergelijkbaar met die bij het maken van een rontgenfoto van buik of longen. Door met een camera (net als bij een röntgenfoto) plaatjes op verschillende tijdstippen te maken kan een goede indruk verkregen worden van de maagontlediging. Op de afdeling wordt aan u uitgelegd door de dietiste hoe u kunt bijhouden wat en hoeveel u drinkt en eet. Hieruit kunnen wij berekenen of u genoeg voeding en vocht binnen krijgt.

**Voor en nadelen van deelname**

Een direct voordeel van deelname is dat er een redelijke kans is dat u eerder kunt eten en drinken, en eerder het ziekenhuis kunt verlaten omdat de complicaties minder kunnen zijn. Een nadeel van deelname is dat u misschien wat meer dorst zal hebben en minder zal plassen, als u in de groep valt met minder infusievloeistof tijdens narcose. Dit laatste wordt beperkt doordat u tijdens de operatie onder narcose bent, en er niets van zal merken. Er wordt t.b.v. het onderzoek, na de operatie, als u op zaal ligt, bij u 2x extra bloed afgenomen in de periode van een week. Dit zal ongeveer 5 ml per keer zijn.

**Privacy**

Ter bescherming van uw privacy worden de onderzoeksgegevens onder code geregistreerd. Alleen de onderzoekers van het AMC kunnen uw identiteit achterhalen. Het kan echter noodzakelijk zijn dat ter controle van de studie aan vertegenwoordigers van daartoe bevoegde overheidsinstanties inzage wordt gegeven in uw medisch dossier. Deze inzage vindt plaats onder verantwoordelijkheid van uw behandelend arts. Deelname aan de studie betekent dat u voor deze inzage toestemming verleent.

**Afzien van deelname**

Uw beslissing tot deelname is geheel vrijwillig. U kunt te allen tijde afzien van deelname aan het onderzoek zonder opgaaf van redenen. Uw besluit heeft uiteraard geen invloed op de zorg die u van uw artsen mag verwachten.Indien u vragen heeft over het onderzoek en dit wil bespreken met een niet bij het onderzoek betrokken arts, dan kunt u contact opnemen met dr Biervliet, telefoon (020) 5669111 sein 63431.

Met vriendelijke groet,

Dr J Hofland, anesthesioloog (sein 8158478)

G van Samkar, anesthesioloog (sein 8158673)

**VERZEKERING**

Overeenkomstig de Wet medisch-wetenschappelijk onderzoek met mensen heeft de AMC Medical Research B.V. voor medisch-wetenschappelijk onderzoek een verzekering afgesloten die door het onderzoek veroorzaakte schade door dood of letsel van de proefpersoon dekt.

* Dit betreft schade die zich tijdens of binnen vier jaar na de deelname aan onderzoek openbaart en gemeld is binnen 4 jaar na beëindiging van de deelname aan het onderzoek.

* Het bedrag waarvoor de verzekering is afgesloten bedraagt € 450.000 per proefpersoon, met een maximum van €3.500.000 voor het gehele onderzoek en € 5.000.000 voor schade ten gevolge van medisch-wetenschappelijk onderzoek die per verzekeringsjaar wordt gemeld.

De verzekering biedt dekking

* voor schade tengevolge van de verwezenlijking van de aan deelname aan het wetenschappelijk onderzoek verbonden risico's waarover men niet schriftelijk is ingelicht;

* voor schade tengevolge van de verwezenlijking van de risico's waarover de deelnemer wél is ingelicht, maar die zich in ernstiger mate voordoet dan is voorzien;

* voor schade tengevolge van de verwezenlijking van de risico's waarover de deelnemer wél is ingelicht, maar die zeer onwaarschijnlijk werd geacht.

De verzekering biedt geen dekking voor schade

* voor schade die het gevolg is van het uitblijven van een vermindering van de gezondheidsproblemen van de proefpersoon, dan wel het gevolg is van de verdere verslechtering van de gezondheidsproblemen, indien de deelname aan het wetenschappelijk onderzoek plaatsvindt in het kader van de behandeling van die gezondheidsproblemen;

* voor schade door aantasting van de gezondheid van de proefpersoon waarvan aannemelijk is dat deze zich ook zou hebben geopenbaard wanneer de proefpersoon niet aan het onderzoek had deelgenomen;

* voor schade tengevolge van deelname aan medisch-wetenschappelijk onderzoek waarbij in de kring van beroepsgenoten gebruikelijke handelingen op het gebied van de geneeskunst met elkaar worden vergeleken en aannemelijk is dat de schade het gevolg is van de toegepaste handelingen;

* voor schade die zich bij een nakomeling van de proefpersoon openbaart als gevolg van een nadelige inwerking van het onderzoek op de proefpersoon of de nakomeling;

* voor schade die het gevolg is van het niet of niet volledig opvolgen van aanwijzingen en instructies door de proefpersoon, indien de proefpersoon daartoe althans in staat is.

De verzekering dekt uitsluitend de schade van natuurlijke personen.

De dekking van specifieke schades en kosten is tot bepaalde bedragen beperkt.

Om aanspraak te kunnen maken op schadevergoeding dient de proefpersoon in geval van vermeende schade als gevolg van het onderzoek dit te melden aan:

naam verzekeraar: Centramed B.A.

adres verzekeraar: Prinses Beatrixlaan 35, 2595 AK 's-Gravenhage.

polisnummer: 620.872.806

Voorts wordt de proefpersoon verzocht dienaangaande contact op te nemen met Prof dr M Hollman (sein 8163932) en mw. M.A. Mooijer (tel 020 – 566 6264)
